# Regulation of lncRNA NUTM2A-AS1 and CCR3 in the Clinical Response to a Plant-Based Diet in Rheumatoid Arthritis: A Pilot Study

**DOI:** 10.3390/nu17111752

**Published:** 2025-05-22

**Authors:** Mario Peña-Peña, Javier González-Ramírez, Elyzabeth Bermúdez-Benítez, José L. Sánchez-Gloria, Luis M. Amezcua-Guerra, Claudia Tavera-Alonso, Carlos A. Guzmán-Martín, Leonor Jacobo-Albavera, Luis H. Silveira-Torre, Laura A. Martínez-Martínez, Fausto Sánchez-Muñoz

**Affiliations:** 1 Sección de Estudios de Posgrado, Escuela Superior de Medicina, Instituto Politécnico Nacional, Mexico City 11340, Mexico; marionutricion2017@gmail.com; 2 Departamento de Fisiología, Instituto Nacional de Cardiología Ignacio Chávez, Mexico City 14080, Mexico; gmcarlos93@gmail.com; 3 Laboratorio de Biología Celular, Facultad de Enfermería, Universidad Autónoma de Baja California, Campus Mexicali, Mexicali 21376, Baja California, Mexico; javier.gonzalez.ramirez@uabc.edu.mx; 4 Departamento de Reumatología, Instituto Nacional de Cardiología Ignacio Chávez, Mexico City 14080, Mexico; elymars@hotmail.com (E.B.-B.); luis_hsil@yahoo.com (L.H.S.-T.); 5 Department of Internal Medicine, Division of Nephrology, Rush University Medical Center, Chicago, IL 60612, USA; jose_sanchez@rush.edu; 6 Departamento de Inmunología, Instituto Nacional de Cardiología Ignacio Chávez, Mexico City 14080, Mexico; lmamezcuag@gmail.com; 7 Laboratorio Central, Instituto Nacional de Cardiología Ignacio Chávez, Mexico City 14080, Mexico; taveramuc@yahoo.com.mx; 8 Laboratorio de Genómica de Enfermedades Cardiovasculares, Instituto Nacional de Médicina Genomica, Mexico City 14610, Mexico; ljacobo@inmegen.gob.mx

**Keywords:** rheumatoid arthritis, long non-coding RNAs, NUTM2A-AS1, CCR3, plant-based diet, inflammatory modulation

## Abstract

**Background/Objectives:** RA is a chronic autoimmune disease characterized by systemic inflammation and progressive joint damage. Plant-based dietary interventions have recently emerged as complementary anti-inflammatory therapy for active RA. However, the molecular anti-inflammatory mechanisms of plant-based dietary patterns in these patients are still poorly understood. Long non-coding RNAs (lncRNAs) have emerged as key regulators of inflammation in chronic diseases. Thus, this study aimed to evaluate the expression of lncRNAs and inflammatory genes in relation to the clinical response to following a plant-based dietary intervention in patients with active RA. **Methods:** A two-phase whole-blood gene expression analysis was conducted for patients with active RA before and after a 14-day plant-based dietary intervention. In the discovery phase, seven patients showing the greatest reduction in disease activity (DAS28-CRP) were selected, and the expression of 84 inflammatory genes and 84 lncRNAs was analyzed using RT^2^ Profiler PCR Array platforms. In the validation phase, by adding 14 patients, we assessed 21 participants. **Results:** NUTM2A-AS1 was the only significantly overexpressed lncRNA in the discovery phase (*p* = 0.0435), while CCR3 was the only inflammatory gene showing significant expression change (*p* = 0.0156). In the validation phase, both NUTM2A-AS1 and CCR3 maintained the same pattern of overexpression, confirming their modulation after the 14-day plant-based dietary intervention (*p* = 0.0131 and *p* < 0.001, respectively). **Conclusions**: This study showed that a 14-day plant-based diet was sufficient to modify the inflammatory circuits in patients with active RA, suggesting a potential dietary-mediated inflammatory modulation mechanism involving NUTM2A-AS1 and CCR3. Further studies are required to validate new hypotheses on the biological significance of the regulation of these transcripts and its clinical implications in RA management.

## 1. Introduction

RA is a chronic autoimmune disease characterized by persistent synovial inflammation, systemic immune activation, and progressive joint damage [1]. These pathological processes contribute to severe pain, disability, and a significant decline in patients’ quality of life. Although disease-modifying antirheumatic drugs (DMARDs) and biologic therapies have improved disease management, many patients experience incomplete responses, adverse effects, or limited access to treatment [2]. This highlights the need for complementary strategies to support pharmacological treatments, such as dietary interventions.

Plant-based diets (PBDs) are dietary patterns that emphasize the consumption of plant-derived foods such as fruits, vegetables, legumes, whole grains, nuts, and seeds, while limiting animal products and processed foods. These diets limit animal-derived foods to varying degrees, including patterns such as lacto-vegetarian, ovo-vegetarian, ovo-lacto-vegetarian, pescatarian, and vegan diets [3]. Current definitions recognize that PBDs do not require the complete elimination of animal products but rather prioritize plant-based sources while allowing flexibility for nutritional adequacy and cultural adaptation [4]. Unlike rigid dietary regimens, these patterns are culturally adaptable and nutritionally diverse. According to UNESCO, plant-based diets are globally sustainable, as they align with traditional food cultures while promoting both health and environmental well-being [5]. This adaptability supports their implementation as complementary strategies in chronic disease management, including for RA.

Emerging evidence suggests that plant-based diets help regulate chronic inflammation, a key driver of RA pathophysiology [6]. These diets are rich in polyphenols, antioxidants, dietary fiber, and omega-3 fatty acids bioactive compounds, known to reduce oxidative stress, modulate immune responses, and suppress pro-inflammatory cytokines [7].

Clinically, adherence to plant-based diets has been associated with reductions in RA disease activity, joint pain, and fatigue [8]. However, the molecular mechanisms behind these improvements, particularly their effects on non-coding RNAs and inflammatory pathways remain largely unexplored.

Long non-coding RNAs (lncRNAs) are transcripts longer than 200 nucleotides that do not encode proteins but regulate gene expression through epigenetic, transcriptional, and post-transcriptional mechanisms [9]. They have emerged as important regulators in autoimmune diseases, including RA, where they modulate immune activation, inflammation, and tissue remodeling [10,11]. Although several lncRNAs have been implicated in RA pathogenesis, their response to dietary interventions remains unexplored.

Similarly, inflammatory gene expression in RA is shaped by complex interactions between immune mediators, environmental triggers, and therapeutic agents [12]. Nutritional factors may influence these molecular pathways, yet few studies have examined how plant-based dietary patterns affect the expression of lncRNAs and inflammation-related genes in patients with RA [13].

To address these gaps, this study aimed to evaluate the expression of lncRNAs and inflammatory genes related to autoimmunity in response to a plant-based dietary intervention in patients with RA.

## 2. Materials and Methods

### 2.1. Patient Recruitment

This study was approved by the Research and Ethics Committee of the Instituto Nacional de Cardiología Ignacio Chávez. The project identification code was 21-1247, with approval granted by the Research Committee on 15 July 2021 and by the Ethics Committee on 22 July 2021. This study adhered to international ethical standards, including the Declaration of Helsinki and Good Clinical Practice guidelines.

The trial was registered retrospectively on ClinicalTrials.gov (identifier: NCT05911880) on 22 June 2023, after the recruitment of participants began on 15 July 2021. Participants were recruited from the rheumatology department of the same institution. Inclusion criteria were adults diagnosed with RA for over one year with mild to moderate disease activity, defined by a DAS28-CRP score between 2.6 and 5.1. Patients were excluded if they had undergone pharmacological treatment changes within three months prior to recruitment or during this study. All participants provided written informed consent before enrolment.

### 2.2. Anthropometric and Biochemical Evaluations

Assessments were performed at baseline and after the 14-day dietary intervention. All anthropometric measurements were conducted in a fasting state and at a consistent time of day. Body composition parameters including body weight, body mass index (BMI), body fat percentage, and visceral fat percentage were measured using an OMRON HBF-514C bioelectrical impedance analyzer (OMRON, Kyoto, Japan). Height was measured with a Seca stadiometer (Seca, Hamburg, Germany), and waist and hip circumferences were obtained using a Lufkin tape (Crescent Lufkin, Saginaw, MI, USA). The waist-to-hip ratio was calculated accordingly. Each measurement was performed twice, and the average was recorded.

Peripheral blood samples were collected using aseptic venipuncture techniques at the same two time points. A total of 4 mL of blood was drawn into EDTA-coated vacutainer tubes for whole-blood separation. On the same day, additional blood samples were sent to the central laboratory for biochemical profiling. Parameters such as glucose, uric acid, triglycerides, total cholesterol, C-reactive protein (CRP), and erythrocyte sedimentation rate (ESR) levels were analyzed using standardized automated methods.

### 2.3. Dietary Intervention

A 14-day personalized, isocaloric plant-based dietary intervention was implemented using traditional Mexican foods. The plan was tailored to each participant’s usual eating habits, which were assessed through three 24 h dietary recalls (two weekdays and one weekend). The total energy expenditure (TEE) was estimated using the Harris–Benedict equation and adjusted for physical activity level to determine individual energy requirements [14]. The diets were designed to meet these needs without restricting caloric intake.

Macronutrient distribution consisted of approximately 57% carbohydrates, 28% fats, and 17% proteins. Plant-based sources such as legumes (beans, lentils), whole grains (corn, oats), seeds, vegetables (squash, cactus), and fruits accounted for the majority of the diet. Approximately 80% of total protein was plant-derived. A limited amount of animal protein (approximately 20%) from culturally accepted sources like panela cheese, eggs, and fish was included to support nutritional adequacy and adherence.

Processed foods and refined sugars were excluded, and foods high in saturated fats were limited to reduce intake of pro-inflammatory components. All meals were based on culturally familiar ingredients, emphasizing traditional staples. This approach ensured the diet reflected participants’ regional food practices and dietary preferences.

Adherence to the dietary plan was monitored through a daily food diary. Compliance was considered acceptable if the reported energy and macronutrient intake ranged between 80% and 120% of the prescribed targets.

### 2.4. Total RNA Isolation and cDNA Synthesis

RNA was extracted from whole blood using a Direct-zol RNA Microprep Kit (Zymo Research, Irvine, CA, USA, Cat. No. R2062). The yield and purity of the RNA were assessed using a NanoDrop 2000 spectrophotometer (Thermo Scientific, Wilmington, DE, USA). The RNA concentration for each sample was quantified to ensure sufficient yield and purity. The quality of the RNA was evaluated by measuring the absorbance ratio at 260/280 nm, consistently recorded at 1.9 ± 0.2. Immediately after extraction, the isolated RNA was reverse-transcribed into complementary DNA (cDNA) using a QuantiTect Reverse Transcription Kit (QIAGEN, Hilden, Germany, Cat. No. 2053), following the manufacturer’s instructions.

### 2.5. Long Non-Coding RNA and Inflammatory Gene Analysis

This study employed a two-phase approach to evaluate the expression of long non-coding RNAs (lncRNAs) and inflammatory genes in patients with RA undergoing a 14-day plant-based dietary intervention.

#### 2.5.1. Discovery Phase

The discovery phase aimed to identify lncRNAs and inflammatory genes responsive to the dietary intervention. Expression levels of 84 lncRNAs and 84 inflammatory genes were assessed at baseline and after 14 days using specific PCR array platforms.

lncRNA analysis was performed using an RT^2^ lncRNA PCR Array Human Inflammatory Response & Autoimmunity (QIAGEN, Cat. No.: 330721, GeneGlobe ID: LAHS-004Z) to profile 84 lncRNAs associated with inflammatory and autoimmune pathways. This array included six housekeeping genes for normalization, three controls for reverse-transcription efficiency, and three controls for PCR quality assurance.

Inflammatory gene analysis was carried out using an RT^2^ Profiler PCR Array Human Inflammatory Response & Autoimmunity (QIAGEN, Cat. No.: 330231, GeneGlobe ID: PAHS-077Z) to analyze 84 inflammatory genes, encompassing cytokines, chemokines, their receptors, and genes involved in cytokine metabolism. This platform also featured six housekeeping genes and six additional controls for validating reverse-transcription and PCR processes.

Seven patients exhibiting the most significant reductions in the Disease Activity Score 28—C-reactive protein (DAS28-CRP) from baseline to 14 days after the intervention were selected for this phase. These patients demonstrated delta DAS28-CRP values ranging from −1.40 to −0.87.

#### 2.5.2. Validation Phase

In the validation phase, the expression of lncRNA NUTM2A-AS1 and the inflammatory gene CCR3, identified as differentially expressed during the discovery phase, was analyzed in the remaining cohort of patients (n = 14) who completed the dietary intervention.

The total RNA was extracted from whole-blood samples using a Direct-zol™ RNA Microprep Kit (Zymo Research, Cat. No. R2062) according to the manufacturer’s protocol. RNA concentration and purity were assessed using a NanoDrop 2000c spectrophotometer (Thermo Scientific, Wilmington, DE, USA), ensuring suitable quality for downstream applications.

Complementary DNA (cDNA) was synthesized from total RNA using a QuantiTect Reverse Transcription Kit (QIAGEN, Venlo, Netherlands, Cat. No. 205311), which included genomic DNA elimination and reverse-transcription steps.

Quantitative real-time PCR (RT-qPCR) was performed using a QuantiNova SYBR Green PCR Kit (QIAGEN, Venlo, Netherlands, Cat. No. 208052). Amplification reactions were carried out using primers specific for NUTM2A-AS1 (RT^2^ lncRNA qPCR Assay for Human NUTM2A-AS1, GeneGlobe ID: LPH04027A-200, Cat. No. 330701), CCR3 (RT^2^ qPCR Primer Assay for Human CCR3, GeneGlobe ID: PPH00613B, Cat. No. 330001), and TNF (RT^2^ qPCR Primer Assay for Human TNF, GeneGlobe ID: PPH00341F, Cat. No. 330001). The housekeeping gene RPLP0 was used as the internal reference for normalization (RT^2^ qPCR Primer Assay for Human RPLP0, GeneGlobe ID: PPH21138F, Cat. No. 330001).

All reactions were performed in duplicate, and relative gene expression was calculated using the ^ΔΔ^Ct method.

### 2.6. Statistical Analysis

The Shapiro–Wilk test was used to assess the normality of the variables. Data are presented as mean ± standard deviation (SD) for normally distributed variables or as median and interquartile range (IQR) for non-normally distributed variables. Categorical variables are reported as frequencies and percentages. Changes between baseline and post-intervention measurements were analyzed using either the paired *t*-test for normally distributed variables or the Wilcoxon signed-rank test for non-normally distributed variables.

Statistical analyses were performed using the Statistical Package for Social Sciences (SPSS, version 26; IBM, Armonk, NY, USA) and GraphPad Prism (version 8.1; GraphPad Software, La Jolla, CA, USA) for computations and data visualization. A *p*-value of <0.05 was considered statistically significant.

## 3. Results

### 3.1. Main Clinical Features of Study Participants

This study initially evaluated 80 patients diagnosed with RA according to the 2010 ACR/EULAR classification criteria. Of these, 51 patients were excluded for not meeting the inclusion criteria: 20 were receiving treatment with coumarin derivatives, 5 had DAS28-CRP scores below 2.6, and 15 had scores above 5.1. Additionally, 11 patients were excluded due to recent changes in their pharmacological treatment.

**Figure 1:**
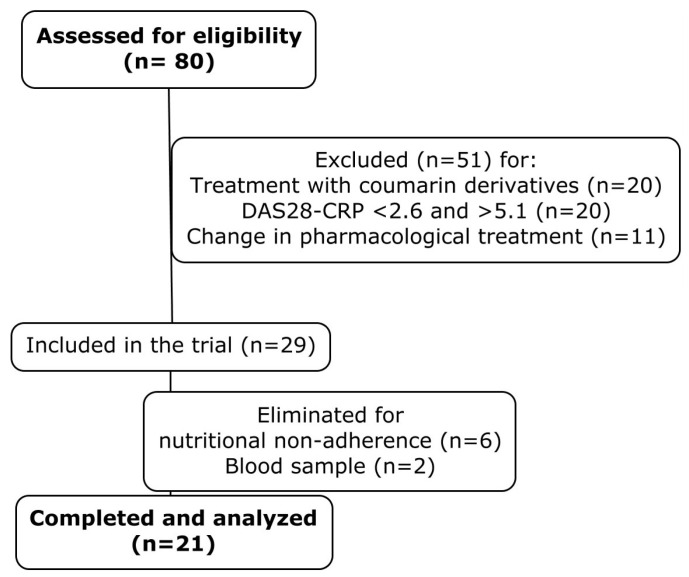
Patient selection and study flowchart. Flowchart summarizing the inclusion, exclusion, and final analysis of study participants following 14-day plant-based dietary intervention.

Subsequently, 29 patients were enrolled in the dietary intervention. However, six were excluded due to non-adherence to the prescribed nutritional plan. Of the remaining 23 patients, the blood samples from 2 were insufficient for analysis. Thus, the final analysis included 21 patients (Figure 1).

The median age of the participants was 56 years (IQR: 48.5–64.0), with women comprising 95% (n = 20) of the cohort. The median body mass index (BMI) was 29.50 kg/m^2^ (IQR: 25.8–33.05), indicating a predominance of overweight and obesity. The median disease duration was 8 years (IQR: 4.5–10.5). Regarding clinical status, the median disease activity measured by DAS28-CRP was 4.04 (IQR: 3.33–4.72), reflecting low to moderate activity.

The comorbid conditions included hypertension in 43%, dyslipidemia and type 2 diabetes mellitus in 19% each, MASLD in 19%, metabolic syndrome in 10%, and pericarditis in 5% of the participants (Table 1).

Regarding treatment, nearly all participants (95.2%) were receiving methotrexate, reflecting a common therapeutic baseline among the study cohort. A substantial proportion were also treated with sulfasalazine (57.1%), hydroxychloroquine (28.6%), and leflunomide (28.6%), indicating the use of combination DMARD therapy in several patients. Importantly, this stable pharmacological regimen being maintained for at least three months prior to and throughout the dietary intervention allowed the evaluation of the clinical and molecular changes attributable to the plant-based diet. Additionally, 28.6% of the patients were prescribed prednisone and paracetamol, suggesting the ongoing management of inflammatory symptoms and pain. The frequent use of folic acid (81.0%) and calcium (61.9%) reflected standard supplementation associated with RA treatment. Among the patients with comorbidities, antihypertensive therapy with losartan (19.0%) and antidiabetic medications such as metformin (42.9%) and pregabalin (33.3%) were also reported, highlighting the metabolic burden commonly observed in this population (Table 2).

### 3.2. Changes in Clinical and Biochemical Parameters Following the Plant-Based Diet

Following the 14-day dietary intervention, significant changes were observed in various clinical and biochemical parameters among the participants. Disease activity, measured by the DAS28-CRP score, showed a notable reduction (*p* < 0.001), indicating a decrease in overall inflammatory activity. Improvements were also detected in body weight (*p* = 0.020), BMI (*p* = 0.001), serum glucose levels (*p* = 0.025), CRP concentrations (*p* = 0.035), and total cholesterol (*p* = 0.005). Additionally, joint assessments revealed significant decreases in both swollen joints (*p* = 0.005) and painful joints (*p* < 0.001) (Table 3).

In contrast, no statistically significant changes were found in the other parameters, such as waist-to-hip ratio, body fat percentage, visceral fat, HDL-C, triglycerides, serum uric acid, or ESR.

### 3.3. Discovery Phase of lncRNAs and Inflammatory Genes Regulated by Clinical Response to a Plant-Based Diet

To explore the molecular changes associated with clinical improvement, the gene expression profiling of the lncRNAs and inflammatory genes was performed in the seven patients who showed the greatest clinical response to the plant-based dietary intervention, as measured by the largest reductions in the DAS28-CRP scores (ΔDAS28-CRP ranging from 1.40 to 0.87).

**Figure 2:**
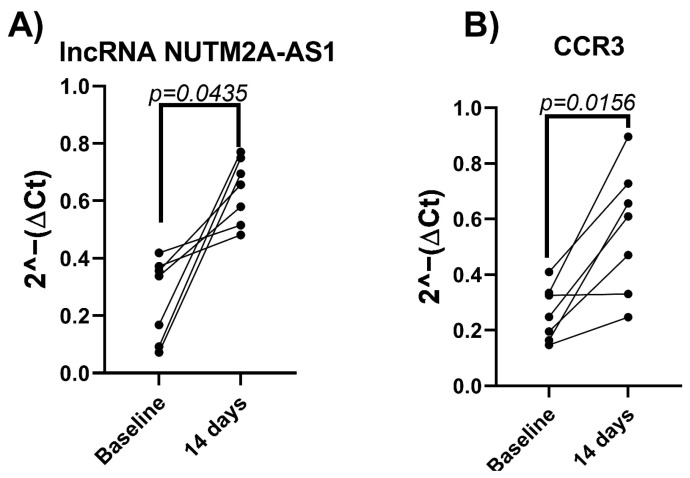
Expression changes in (**A**) NUTM2A-AS1 and (**B**) CCR3 in the discovery phase (n = 7). Expression levels were measured in whole-blood samples collected from the seven patients with RA who demonstrated the greatest clinical improvement based on ΔDAS28-CRP scores following a 14-day plant-based dietary intervention. Gene expression was quantified using the 2^−(ΔCt)^ method and is presented on a log scale. Each line represents a paired measurement from an individual participant. Significant upregulations of NUTM2A-AS1 (*p* = 0.0435) and CCR3 (*p* = 0.0156) were observed after the intervention. Statistical comparisons were performed using the Wilcoxon signed-rank test.

Among the 84 long non-coding RNAs (lncRNAs) analyzed, NUTM2A-AS1 emerged as the only differentially expressed lncRNA following the 14-day intervention. A significant increase in its expression was observed after the intervention compared to baseline levels (*p* = 0.0435) (Figure 2A).

Regarding inflammatory gene expression, only CCR3 exhibited a significant change in expression after the dietary intervention (*p* = 0.0156) (Figure 2B).

### 3.4. Validation Phase of Consistent Expression Patterns of NUTM2A-AS1 and CCR3 in the Remaining Cohort

In the validation group (n = 14), the median DAS28-CRP was 3.84 (IQR: 3.28–4.21) at baseline and decreased to 3.31 (IQR: 2.90–3.54) after 14 days of the dietary intervention.

To confirm the findings from the discovery phase, the expressions of NUTM2A-AS1 and CCR3 were analyzed in the remaining 14 participants who completed the dietary intervention.

**Figure 3:**
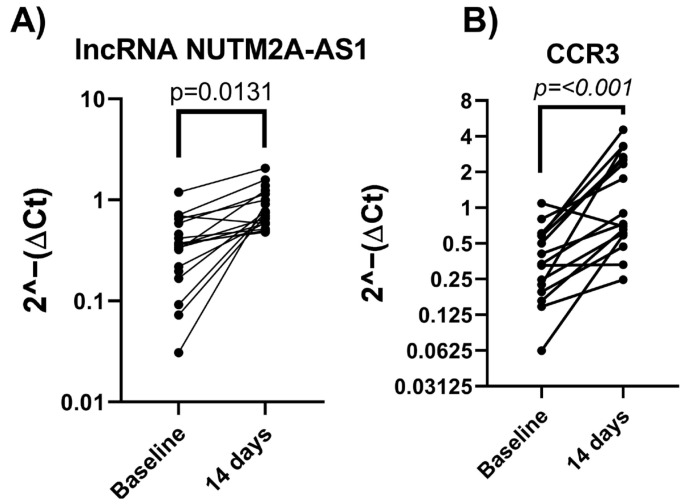
Validation of NUTM2A-AS1 and CCR3 expressions in the remaining patients with RA (n = 21). Paired expression analysis of (**A**) lncRNA NUTM2A-AS1 and (**B**) CCR3 in peripheral whole-blood samples before and after a 14-day plant-based dietary intervention. Gene expression levels were calculated using the 2^−(ΔCt)^ method and are plotted on a log scale. Each line represents an individual patient. Statistical comparisons were performed using the Wilcoxon signed-rank test for paired non-parametric data. NUTM2A-AS1 showed a significant increase in expression (*p* = 0.0131), and CCR3 exhibited a robust upregulation (*p* < 0.001).

The validation phase confirmed the significant post-intervention upregulation of NUTM2A-AS1 (*p* = 0.0131) (Figure 3A). Likewise, CCR3 expression also exhibited a consistent and significant increase (*p* < 0.001) (Figure 3B). These results support the findings from the discovery phase and reinforce the potential of these molecules as markers of dietary modulation of inflammation in RA.

**Figure 4:**
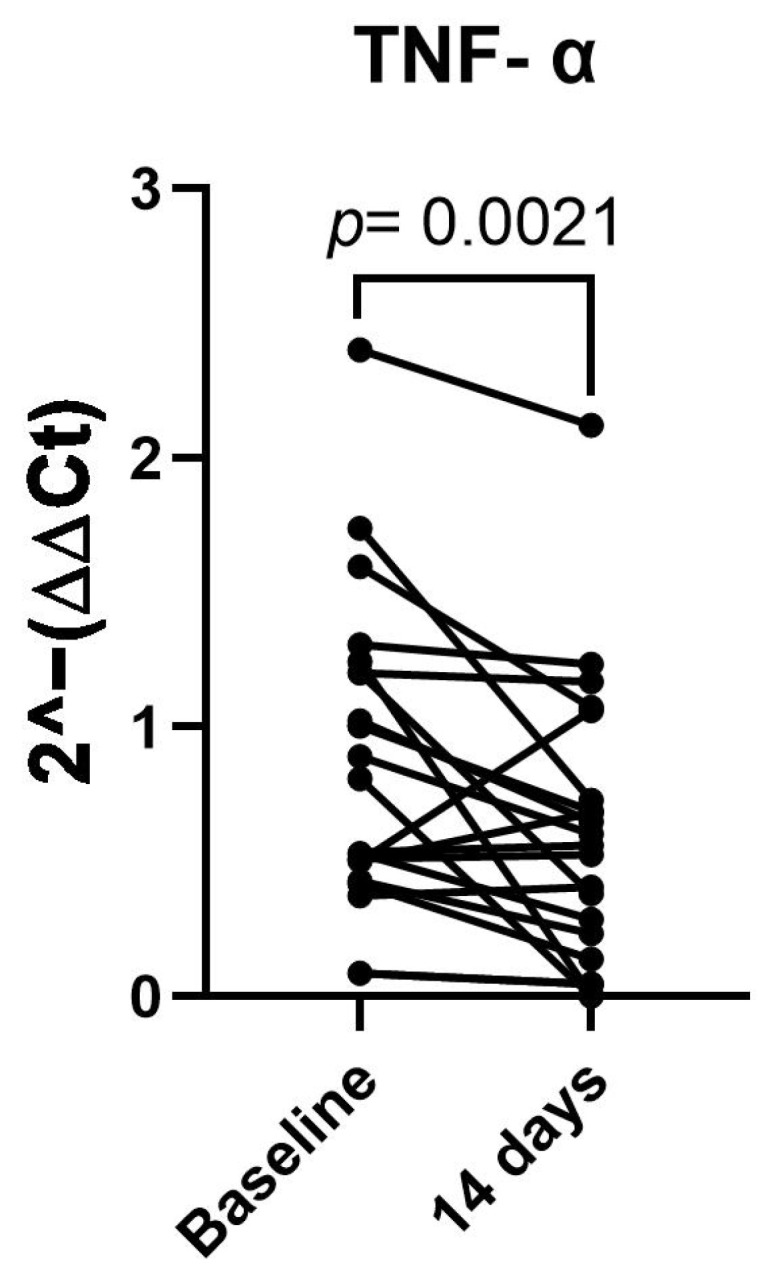
TNF-α expression before and after 14 days of dietary intervention in whole-blood samples from 21 patients. Data are presented as 2^–(ΔΔCt)^.

Finally, to explore whether the observed changes were related to systemic inflammation, the TNF-α expression was analyzed in the whole-blood samples from all participants. A significant reduction in the TNF-α levels was observed after 14 days of the dietary intervention (*p* = 0.0021), indicating a general decrease in inflammatory status (Figure 4).

## 4. Discussion

This study explored the clinical and molecular effects of a short-term plant-based dietary intervention in patients with active RA. Improvements were observed in disease activity, inflammatory markers, metabolic parameters, and the expressions of NUTM2A-AS1 and CCR3.

Regarding the two-phase gene expression analysis, we observed a significant upregulation of the lncRNA NUTM2A-AS1 following the plant-based dietary intervention in patients with RA (*p* = 0.0131). To the best of our knowledge, this is the first report describing diet-induced modulation of NUTM2A-AS1 in this context. This finding contrasts those of earlier studies that associated elevated NUTM2A-AS1 expression with pro-inflammatory activity in other pathological conditions [15].

In this sense, we observed a consistent upregulation of the long non-coding RNA NUTM2A-AS1 following a short-term plant-based dietary intervention in patients with active RA. While NUTM2A-AS1 has not been previously studied in the context of rheumatoid arthritis or dietary modulation, its regulatory potential in inflammation has been described in other disease models, particularly cancer. Mechanistically, NUTM2A-AS1 has been shown to activate the Wnt/β-catenin signaling pathway, a central regulator of inflammation, proliferation, and immune cell differentiation [16]. Furthermore, it has been implicated in the upregulation of pro-inflammatory cytokines such as IL-6, with studies showing that its suppression, e.g., by curcumin, reduces IL-6 expression and increases apoptosis in hepatocellular carcinoma cells [15].

Beyond signaling pathways, NUTM2A-AS1 also appears to function as a competing endogenous RNA (ceRNA) by sponging regulatory microRNAs, including miR-183-5p and miR-376a-3p, thereby modulating target gene expression related to inflammation and survival [17,18]. In neuroblastoma, it has been shown to stabilize the immune checkpoint molecule B7-H3, preventing its degradation and supporting immune evasion [19]. These findings collectively suggest that NUTM2A-AS1 has broader immunoregulatory effects beyond oncogenic contexts, potentially influencing the immune activation and resolution phases.

The upregulation of NUTM2A-AS1 observed in our cohort may thus represent a context-dependent and potentially compensatory immune response to the anti-inflammatory dietary environment. This interpretation is supported by prior reports emphasizing that lncRNAs are highly cell-type- and condition-specific, often reflecting microenvironmental cues more sensitively than protein-coding genes [20,21]. Whether NUTM2A-AS1 acts directly to modulate inflammatory gene networks in RA or represents a biomarker of dietary immune adaptation remains to be fully clarified. Nevertheless, our findings introduce NUTM2A-AS1 as a promising target for future studies on the transcriptomic response to dietary interventions in autoimmune disease.

On the other hand, our study identified a significant upregulation of CCR3 expression following the 14-day plant-based dietary intervention in patients with active RA (*p* < 0.001). While CCR3 is classically viewed as a pro-inflammatory chemokine receptor, predominantly expressed on eosinophils and Th2 lymphocytes, recent evidence suggests a more nuanced and context-dependent role, particularly in chronic and metabolically influenced inflammation. In RA, CCR3 has been detected in various immune cell subsets, including fibroblast-like synoviocytes (FLSs) [22], monocytes [23], and peripheral T cells [24], where its activation has been linked to the enhanced production of matrix metalloproteinases (MMPs) and the increased migratory behavior of inflammatory cells [22].

Experimental models have shown that the pharmacological inhibition of CCR3 can attenuate leukocyte infiltration and joint inflammation [25], and similar findings have been reported in eosinophilic inflammation [26]. However, other evidence suggests that CCR3 expression is also associated with immune adaptation, particularly in response to dietary and metabolic stimuli. In atherosclerosis-prone mice, CCR3 deletion exacerbated vascular damage under hyperlipidemic conditions, indicating that CCR3 may play a role in maintaining the immune balance depending on the inflammatory context [27]. Similarly, in models of MAFLD, CCR3+ monocytes were involved in tissue rebalancing and the resolution of hepatic inflammation [28].

Importantly, the dietary modulation of CCR3 expression has been documented. Grape seed proanthocyanidins downregulated CCR3 in human peripheral blood mononuclear cells [29], while, in contrast, a 14-day vegan diet in healthy adults upregulated the gene signatures associated with monocytes, neutrophils, and type I interferon pathways, supporting the concept of transcriptional immune adaptation in response to diet [30]. Therefore, the increase in CCR3 observed in our study may not directly signify heightened inflammatory activity but rather a transient immune reprogramming toward a different regulatory state as part of the host adaptation to a nutrient-dense, plant-based diet.

Although the precise functional outcome of CCR3 upregulation in this context remains to be determined, our data—when considered alongside NUTM2A-AS1 upregulation—suggest that dietary interventions influence immune signaling at the transcriptomic level. These findings support the hypothesis that specific dietary patterns can rewire inflammatory gene networks, warranting further mechanistic studies to clarify the cellular sources, ligand engagement, and downstream effects of CCR3 in response to nutritional modulation in RA [22,24,25,26,27,28,29].

Some limitations should be acknowledged. This study offers early insights into the clinical and transcriptomic effects of a 14-day plant-based dietary intervention in patients with active rheumatoid arthritis. Despite the short duration and small sample size, significant improvements in disease activity and gene expression were observed. The lack of a control group is a limitation, but the self-controlled design with stable pharmacological regimens minimized variability, making it appropriate as a pilot study. Notably, CCR3 upregulation correlated with reduced joint inflammation, suggesting clinical relevance, while NUTM2A-AS1 changes did not correlate with the clinical parameters. The functional roles of both transcripts remain unclear and warrant further investigation. Future research should include longer interventions, randomized designs, and mechanistic studies to understand the dietary modulation of the immune responses in RA.

## 5. Conclusions

This study provides preliminary evidence that a short-term plant-based dietary intervention influences clinical outcomes and of the expression of the molecular markers involved in inflammation and autoimmunity in patients with active RA. Improvements in disease activity, metabolic parameters, and inflammatory markers were observed without changes in pharmacological treatment, suggesting a contribution of the dietary intervention.

At the molecular level, the consistent upregulation of NUTM2A-AS1 and CCR3 across both the discovery and validation phases highlights these molecules as potential biomarkers of the host transcriptomic response to plant-based nutrition. These findings offer a basis for future studies aimed at understanding the mechanisms through which dietary patterns influence immune regulation in chronic inflammatory diseases.

Further research with larger cohorts and over longer intervention periods is needed to confirm these observations and to determine the clinical utility of NUTM2A-AS1 and CCR3 as markers of the response to nutritional interventions in RA.

## Figures and Tables

**Table 1:** Baseline demographic and clinical characteristics.

	Patients with RA (n = 21)
Age in years	56 (48.5-64)
Female sex, n (%)	20 (95)
BMI (kg/m^2^)	29.50 (25.8–33.05)
Disease duration (years)	8 (4.5–10.5)
DAS28-CRP	4.04 (3.33–4.72)
Hypertension n (%)	9 (43)
Dyslipidemia n (%)	4 (19)
Type 2 diabetes mellitus n (%)	4 (19)
MASLD n (%)	4 (19)
Metabolic syndrome n (%)	2 (10)
Pericarditis n (%)	1 (5)

Data are presented as median (interquartile range) for continuous variables and as frequency (percentage) for categorical variables. DAS28-CRP: Disease Activity Score-28 based on C-reactive protein; BMI: Body mass index; MASLD: metabolic-dysfunction-associated steatotic liver disease.

**Table 2:** Pharmacological treatment was maintained during the 3 months prior to and throughout the 14-day plant-based dietary intervention in patients with RA.

Classification	Medication	n (%)
Disease-Modifying antirheumatic drugs (DMARDs)	Methotrexate	20 (95.2%)
	Sulfasalazine	12 (57.1%)
	Hydroxychloroquine	6 (28.6%)
	Leflunomide	6 (28.6%
Supplements	Folic acid	17 (81.0%)
	Calcium	13 (61.9%)
Glucocorticoids	Prednisone	6 (28.6%)
Analgesics/anti-inflammatory	Paracetamol	6 (28.6%)
Hypertension (HAS)	Losartan	4 (19.0%)
Diabetes	Metformin	9 (42.9%)
	Pregabalin	7 (33.3%)

Data are expressed as absolute frequency (n) and percentage (%). All patients maintained a stable pharmacological regimen for at least three months prior to this study and throughout the 14-day plant-based dietary intervention.

**Table 3:** Clinical and biochemical parameters before and after the plant-based diet in patients with RA.

	Baseline	14 Days	*p*
Weight (kg)	65.50 (60.75–83.05)	64.70 (59.25–83.65)	**0.02**
Waist-to-hip radio	0.88 (0.84–0.94)	0.89 (0.84–0.92)	0.387
BMI (kg/m^2^)	29.50 (25.8–33.05)	29.2 (25.15–32.75)	**0. 001**
% Body fat	44.4 (37.1–48.05)	45 (37.25–49.4)	0.736
%Visceral fat	10 (7.5–12)	10 (7–12.5)	0.052
Serum glucose (mg/dL)	92 (82.5–104)	87 (80–99)	**0.025**
Serum uric acid (mg/dL)	4.91 (3.9–5.81)	4.98 (4.19–5.61)	0.575
Total cholesterol (mg/dL)	180 (144–211)	155 (141–199)	**0.005**
HDL-C (mg/dL)	47.5 (41.65–60.4)	46.5 (41.1–56.8)	0.487
Triglycerides (mg/dL)	134 (106–174)	130 (107.5–176)	0.214
CRP (mg/L)	5.61 (3.38–8.96)	4.78 (2.35–7.4)	**0.035**
ESR (mm/h)	17 (7.5–33.5)	15 (8–25)	0.061
Swollen joints	5.00 (3–8)	3 (1.5–4.5)	**0.005**
Painful joints	7 (2.5–8)	3 (1–3.5)	**<0.0001**
DAS28-CRP	4.04 (3.33–4.72)	3.43 (2.92–3.6)	**<0.0001**

Results are expressed as median (interquartile range). Differences between baseline and day-14 values were analyzed using the Wilcoxon signed-rank test. BMI = body mass index; HDL-C = high-density lipoprotein cholesterol; CRP = C-reactive protein; ESR = erythrocyte sedimentation rate; DAS28-CRP = Disease Activity Score-28 based on C-reactive protein. Significant *p* value in bold.

## Data Availability

Raw data are available upon reasonable request from the corresponding author.

## Abbreviations

The following abbreviations are used in this manuscript:

RA	Rheumatoid arthritis
DAS28-CRP	Disease Activity Score 28 using C-reactive protein
DMARDs	Disease-modifying antirheumatic drugs
BMI	Body mass index
MASLD	Metabolic-dysfunction-associated steatotic liver disease
CRP	C-reactive protein
ESR	Erythrocyte sedimentation rate
EDTA	Ethylenediaminetetraacetic acid
TEE	Total energy expenditure
cDNA	Complementary DNA
lncRNA	Long non-coding RNA
RT-qPCR	Quantitative real-time polymerase chain reaction
RPLP0	Ribosomal protein lateral stalk subunit P0
SD	Standard deviation
IQR	Interquartile range
SPSS	Statistical Package for the Social Sciences
NUTM2A-AS1	NUT family member 2A antisense RNA 1
CCR3	C-C motif chemokine receptor 3
IL-6	Interleukin 6
TNF-α	Tumor necrosis factor alpha
NF-κB	Nuclear factor kappa-light-chain-enhancer of activated B cells
Th2	T helper 2
MAFLD	Metabolic-associated fatty liver disease
